# Exogenously Applied Ascorbic Acid-Mediated Changes in Osmoprotection and Oxidative Defense System Enhanced Water Stress Tolerance in Different Cultivars of Safflower (*Carthamus tinctorious* L.)

**DOI:** 10.3390/plants9010104

**Published:** 2020-01-14

**Authors:** Ayesha Farooq, Shazia Anwer Bukhari, Nudrat A. Akram, Muhammad Ashraf, Leonard Wijaya, Mohammed Nasser Alyemeni, Parvaiz Ahmad

**Affiliations:** 1 Department of Biochemistry, Government College University, Faisalabad 38040, Pakistan; afarooq@gmail.com; 2 Department of Botany, Government College University, Faisalabad 38040, Pakistan; bukharisa@gmail.com; 3 Department of Botany, University of Agriculture, Faisalabad 38040, Pakistan; ashrafbot@yahoo.com; 4 Botany and Microbiology Department, College of Science, King Saud University, Riyadh 11451, Saudi Arabia; leon077@gmail.com (L.W.);; 5 Department of Botany, S.P. College, Srinagar 190001, India

**Keywords:** water stress, safflower, ascorbic acid, lipid peroxidation, antioxidants, osmoprotectants

## Abstract

The present study was conducted to examine the effect of exogenously applied ascorbic acid (AsA) on osmoprotectants and the oxidative defense system in four cultivars (16171, 16183, 16207 and 16246) of safflower under well-watered and water deficit conditions. Water stress (60% field capacity) significantly decreased the shoot and root fresh and dry weights, shoot and root lengths and chlorophyll contents in all four safflower cultivars, while it increased the leaf free proline, total phenolics, total soluble proteins, hydrogen peroxide content and activities of catalase, superoxide dismutase and peroxidase enzymes. Foliar-applied (100 mg L^−1^ and 150 mg L^−1^) ascorbic acid caused a marked improvement in shoot and root fresh and dry weights, plant height, chlorophyll and AsA contents as well as the activity of peroxidase (POD) enzyme particularly under water deficit conditions. It also increased the accumulation of leaf proline, total phenolics, total soluble proteins and glycine betaine (GB) content in all four cultivars. Exogenously applied AsA lowered the contents of MDA and H_2_O_2,_ and the activities of CAT and SOD enzymes. Overall, exogenously applied AsA had a positive effect on the growth of safflower plants under water deficit conditions which could be related to AsA-induced enhanced osmoprotection and regulation of antioxidant defense system.

## 1. Introduction

Drought stress can negatively affect the yield of various foods and crops, by influencing their physio-biochemical features [1,2]. Enhanced food demand for the growing population and water shortage due to lower rainfall and other climatic changes may cause famine [3]. Drought stress can directly affect photosynthesis, development, nutrient uptake/accumulation and osmotic adjustment, ultimately causing a marked suppression in crop yield [1,4,5]. Water deficit interferes with photosynthesis either by altering the photosynthetic metabolic pathway or by inducing the over-generation of oxidative stress. Decline in the rate of photosynthesis is then affected by reduced CO_2_ diffusion [6]. Photosynthetic pigments and thylakoid membranes are also denatured by water deficit conditions [7]. More importantly, hydrogen peroxide, superoxide, peroxide and hydroxyl radicals are produced during the photorespiratory reaction, which can effectively damage the biological membranes and cellular biomolecules [3,8]. Many enzymatic and non-enzymatic antioxidants accumulate to a considerable level in stressed plants to counteract ROS [9]. Non-enzymatic antioxidants like ascorbic acid, β-carotene, tocopherols and their derivatives as well as enzymatic antioxidants like peroxidase, ascorbate peroxidase (APX), superoxide dismutase (SOD), and catalase (CAT) execute the antioxidant capacity [9,10].

One of the prominent non-enzymatic antioxidants is ascorbic acid (AsA) which acts as a powerful antioxidant in addition to its critical role in several key metabolic processes [11]. Ascorbic acid is commonly known as vitamin C and it is well known to regulate stress tolerance in plants as reported in a number of studies, e.g., pea [12], canola [13] and maize [14] etc. Furthermore, it acts as a co-factor for enzymes that work in various metabolic pathways [15]. Moreover, exogenously applied ascorbic acid is believed to be very effective in protecting proteins and lipids in plants exposed to water deficit and higher saline regimes [11,16]. By increasing the activities of CAT, SOD, POD enzymes and proline content, minimizing the H_2_O_2_ production and elevating the levels of phenolics, ascorbic acid makes a plant resistant to different abiotic stresses [11]. 

Safflower (*Carthamus tinctorius* L.) is a potential oil-seed crop as it yields about 32–40% oil with all vital nutrients [17,18]. Its plant parts have a considerable utilization in herbal medicines. For example, its leaf extract is used for treating hyperlipidemia and cardiovascular disorder [19]. Its flowers are used to extract natural dye, which is widely used in food as well as textile industry. Thus, safflower is a multipurpose crop. It has been noticed that safflower yield declines markedly in countries with large cultivating areas but with less rainfall [1]. Thus, there is a need to identify or develop drought-tolerant cultivars of this potential crop for successfully utilizing the drought-hit areas. But for a successful selection/screening of crop cultivars for drought tolerance, there is a need to identify potential selection criteria for physio-biochemicals.

Thus, the present study was aimed to assess the effect of water deficit stress-induced oxidative stress on some cultivars of safflower. The production of some key reactive oxygen species and how they are counteracted by different metabolic antioxidants including both enzymatic and non-enzymatic ones was assessed. It is well known that ascorbic acid is one of the potential non-enzymatic antioxidants and a potential plant growth regulator, so the present study was conducted to determine to what extent exogenous application of AsA could regulate the oxidative defense system in safflower plants under water deficit conditions.

## 2. Materials and Methods

### 2.1. Experimental Conditions 

In the present study, four differentially water-stress-tolerant cultivars, 16171, 16183, 16207 and 16246 of safflower (*Carthamus tinctorius* L.) were grown under well-watered and water-deficit conditions. A completely randomized experiment was designed with four replicates comprising a total of 96 pots (water stress (2) × cultivars (4) × AsA treatments (3) × replicates (4) = 96). Seeds of all four cultivars were grown in the plastic pots, 25 cm in depth and 12 cm in radius, under two water regimes (100% (control) and 60% field capacity (water stress)). Each pot contained 8 kg of sandy-loam soil. In each pot, eight seeds were sown. One week after sowing, the germinated seedlings were thinned to maintain five plants in each pot. Soil moisture contents were maintained daily. Three weeks after germination, the two water treatments (100% field capacity and 60% field capacity) were initiated. For water deficit stress treatments, regular measurements of the weights of the pots was made daily to keep the soil moisture content at the desired level. After two weeks of water stress all the experimental units were then subjected to foliar spray of three concentrations (control (0 mg/L), 100 and 150 mg/L) of ascorbic acid. After two weeks of application of the AsA, four plants per replicate were harvested and washed with distilled water. Data for root and shoot lengths, and root and shoot fresh and dry weights were recorded after forty-nine days of seed germination. For measuring dry weights of roots and shoots, the plant samples were kept in an oven at 70 °C for 3 days and then their dried weights were measured.

### 2.2. Physiological Parameters

Before harvesting, the following physio-biochemical attributes were measured:

#### 2.2.1. Chlorophyll Contents 

A fresh leaf sample (0.5 g) from each treatment was homogenized in 10 mL acetone (80%). All the samples were centrifuged at 15,000 *rpm* for 15 min and kept at 4 °C overnight following Arnon [20]. Then the absorbance of the extracts was measured using UV-visible spectrophotometer (Model Hitachi-U 2001, Tokyo, Japan) at 663 and 645 nm. Chlorophyll *a* and *b* contents were calculated by the following formulae:

Chlorophyll *a* (mg/g fresh weight) = [12.7(OD 663) − 2.69(OD 645)] × V/1000 × W (V, volume and W, Weight)

Chlorophyll *b* (mg/g fresh weight) = [22.9(OD 663) − 4.68(OD 645)] × V/1000 × W

#### 2.2.2. Hydrogen Peroxide (H_2_O_2_)

H_2_O_2_ was determined using the method outlined by Velikova, et al. [21]. A proportion of fresh leaf sample (0.5 g) was homogenized in a chilled mortar and pestle with 5 mL of 0.1% trichloroacetic acid (TCA). After filtration, 1.0 mL supernatant was mixed with 0.5 mL phosphate buffer and 1 mL of 1 M potassium iodide. The sample mixtures were vortexed thoroughly and their absorbance read at 390 nm using a spectrophotometer. H_2_O_2_ was calculated from a standard curve developed by using tannic acid as standard. 

#### 2.2.3. Ascorbic Acid (AsA)

A fresh leaf sample (0.5 g) was homogenized in 10 mL of TCA (6%). Then 4 mL of the extract were mixed with 2 mL of dinitrophenyl hydrazine and then 1 drop of thiourea was added to the mixture. The mixture was boiled for 15 min and then allowed to cool to room temperature. Five mL of 80% H_2_SO_4_ were added to the mixture. Absorbances of all treated samples were read at 530 nm following Mukherjee and Choudhuri [22] and compared with standard curve drawn by using ascorbic acid ranges from 10–100 mg L^−1^. 

#### 2.2.4. Malondialdehyde (MDA)

For the measurement of MDA, the method of Cakmak and Horst [23] was followed. A fresh leaf sample (0.5 g) was ground in a chilled mortar and pestle containing 5 mL of 1% (*w*/*v*) TCA. The mixture was centrifuged (HERMLE Z216-MK; Microcentrifuge, Wehingen, Germany) at 15,000 *rpm* for 10 min. Took mL of the supernatant, 4 mL of 0.5% thiobarbituric acid (TBA) were added. The mixture was boiled at 95 °C and cooled. The absorbance of all treated samples was recorded at 532 and 600 nm using a spectrophotometer. The level of TBA was calculated using the absorption co-efficient, 155 mmol cm^−1^.

MDA = ∆ (OD532 − OD600)/1.56 × 10^5^

#### 2.2.5. Total Phenolics

To determine the total phenolic contents the method of Julkunen-Tiitto [24] was followed. Each (0.1 g) of the fresh leaf samples was ground in 5 mL of 80% acetone and then centrifuged at 10,000 *rpm* for 10 min. To 0.1 mL of the supernatant, 1 mL of the Folin-Ciocalteu’s phenol reagent was added. The mixture was shaken vigorously, 5 mL of 20% sodium carbonate was added, and the final volume was made up to 10 mL by adding distilled water. The absorbance of all treated samples was read at 750 nm.

#### 2.2.6. Glycinebetaine (GB)

A fresh leaf (0.5 g) sample was taken and homogenized with 5 mL of 0.5% toluene, filtered it and the supernatant stored at 4 °C. After 12 h, 1 mL of the filtrate was mixed with 1 mL of sulfuric acid. Then, 0.5 mL of this mixture was taken in a test tube and added 200 μL of potassium tri-iodide to it. After cooling on ice, 2.8 mL of chilled double deionized water and 5 mL of 1, 2-di-chloroethane were added to it. The absorbance of the lower layer (organic) was recorded at 365 nm and GB calculated using standard curve following the protocol of Grieve and Grattan [25].

#### 2.2.7. Proline

A fresh leaf (0.5 g) was ground well with 5 mL of sulfosalicylic acid (3% *w*/*v*). The extract was filtered and 2 mL acid ninhydrin solution were added to 2 mL of the filtrate. The absorbance of the colored solution was read at 520 nm following Bates, et al. [26]. Toluene was used as a blank and proline was calculated as:

μmol proline g^−1^ FW = μg proline mL^−1^ × mL of toluene/115.5)/(sample wt (g)).

#### 2.2.8. Antioxidant Enzymes

For enzyme extraction, a sample (0.5 g) of fresh leaf was homogenized with 10 mL of sodium phosphate buffer (pH 7.8). The extract supernatant obtained after centrifugation for 15 min at 15,000 *rpm* was preserved at −20 °C in an ultra-low freezer. Activities of superoxide dismutase (SOD), peroxidase (POD) and catalase (CAT) enzymes were recorded as follows:

For measuring the activity of SOD enzyme, a reaction mixture was prepared (0.4 mL H_2_O + 0.25 mL of phosphate buffer + 0.1 mL methionine + 0.1 mL triton-X + 0.5 mL NBT + 0.5 mL enzyme extract + 0.5 mL riboflavin) according to van Rossum, et al. [27]. Then, the mixture was kept in the light for 15 min and the absorbance recorded at 560 nm. A method proposed by Chance and Maehly [28] was used to determine the activity of POD enzyme. According to this method, 1.8 mL phosphate buffer (pH 7.8) was added to 0.1 mL 0.5% guaiacol, 0.1 mL H_2_O_2_ (0.5%) and enzymes extract (0.1 mL). Then, the absorbance was recorded at 400 nm for 3 min at intervals of 30 sec using a spectrophotometer. To determine the activity of CAT enzyme, we followed the method proposed by Luck [29]. We took 0.1 mL of plant extract, mixed it with 1 mL of 5.9 mM H_2_O_2_ and 1.9 mL of 50 mM phosphate buffer. The absorbance was recorded at intervals of 20 s for three minutes at 240 nm. A change of 0.01 A240 Units min^−1^ was considered to be equal to 1 Unit of CAT activity. SOD, POD and CAT activities were then calculated and expressed on mg^−1^ total soluble protein (TSP).

#### 2.2.9. Statistical Analysis

A three-way analysis of variance (ANOVA) of data for all attributes was carried out using CoStat V6. 303 to test the effects of foliar spray of ascorbic acid on safflower under water deficit conditions. 

## 3. Results

**Figure 1:**
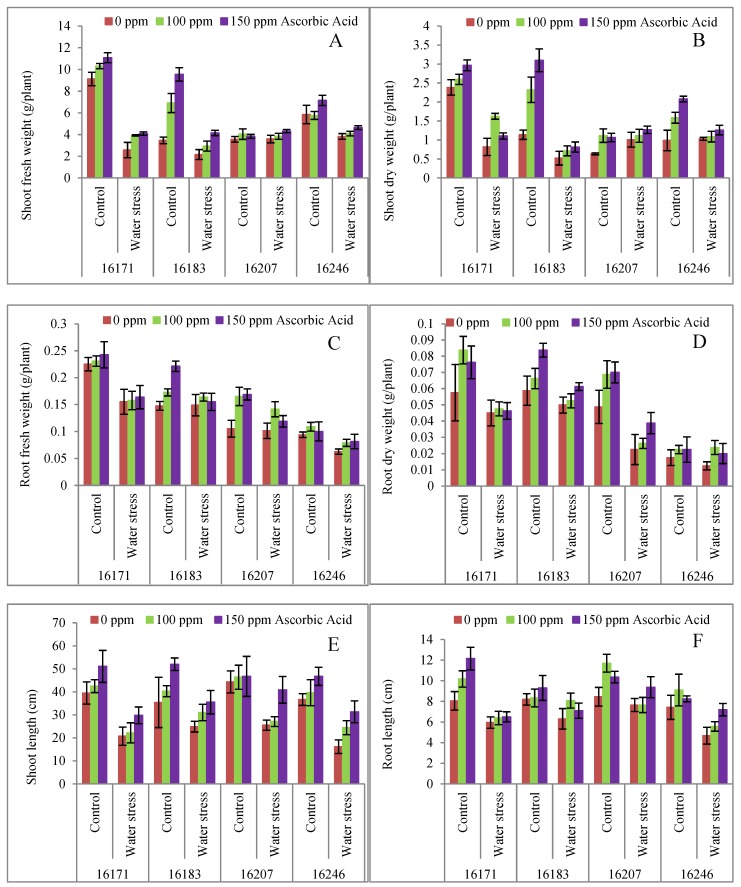
Shoot fresh (**A**) and dry (**B**) weights, root fresh (**C**) and dry (**D**) weights, shoot (**E**) and root (**F**) lengths, of four cultivars of safflower (*Carthamus tinctorius* L.) foliarly treated with ascorbic acid subjected to water stress conditions (mean ± S.E.).

Water stress (60% field capacity) significantly (*p* ≤ 0.001) decreased the shoot and root fresh and dry weights of all four (16171, 16183, 16207 and 16246) cultivars of safflower (Table 1; Figure 1A–D). Foliar-applied (100 and 150 mg L^−1^) ascorbic acid (AsA) significantly improved shoot and root biomass of safflower cultivars under water deficit conditions except for cultivar 16207 wherein the biomass remained unchanged. A significant (*p* ≤ 0.001) difference was observed among all safflower cultivars and of all cultivars, the growth of cultivars 16171 and 16183, was greatly improved by exogenously applied ascorbic acid under water deficit conditions.

Shoot and root lengths were also significantly (*p* ≤ 0.001) suppressed by the water stress (Table 1; Figure 1E,F). However, foliar applied ascorbic acid improved the shoot as well as root lengths of all cultivars of safflower under water deficit regimes. The response of all safflower cultivars remained almost unchanged in terms of shoot length, while root length was found to be increased significantly in cultivars 16183 and 16207 particularly under water deficit conditions. 

**Figure 2:**
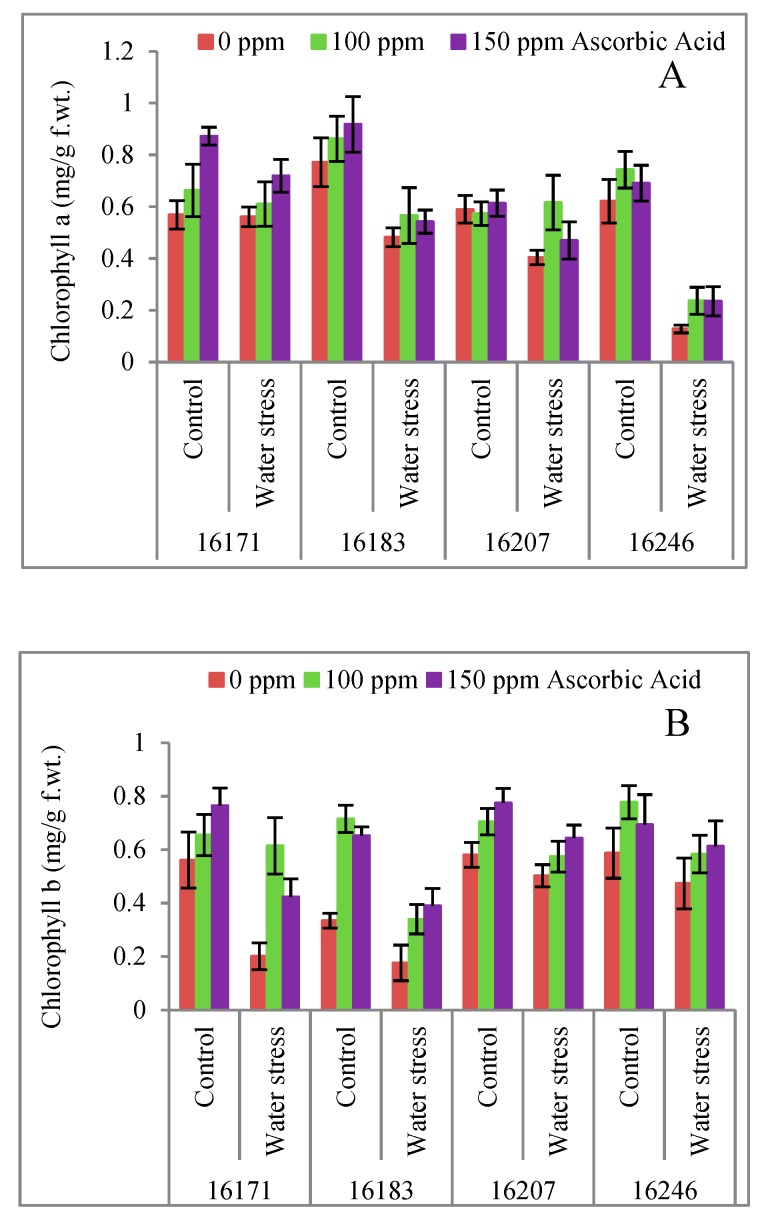
Chlorophyll a (**A**), chlorophyll b (**B**) contents of four cultivars of safflower (*Carthamus tinctorius* L.) foliarly treated with ascorbic acid subjected to water stress conditions (mean ± S.E.).

Chlorophyll *a*, chlorophyll *b* contents decreased significantly (*p* ≤ 0.001) under water stress. Ascorbic acid significantly (*p* ≤ 0.001) improved chlorophyll (*a*, *b*) contents under both water regimes. Of all safflower cultivars, cvs. 16171, 16207 followed by 16246 had higher chlorophyll *a* and *b* contents under water stress conditions (Table 1; Figure 2A,B).

**Figure 3:**
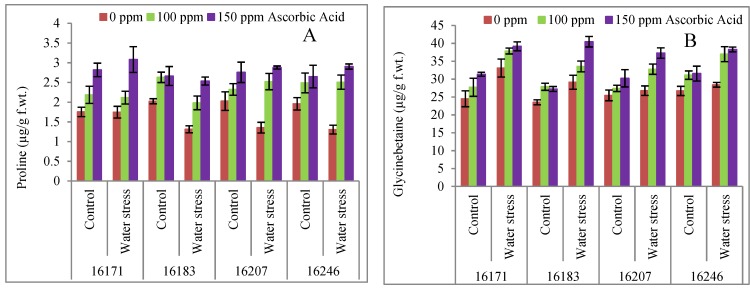

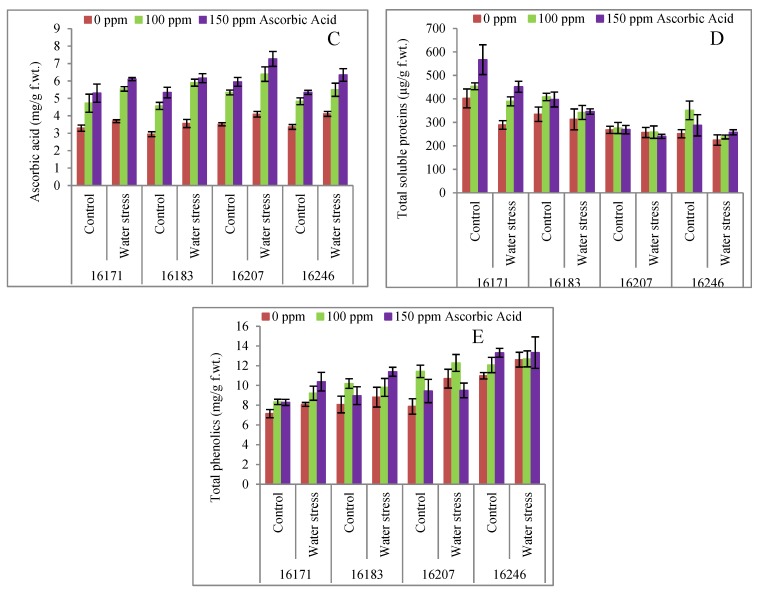
Proline (**A**), glycine betaine (**B**), ascorbic acid (**C**), total soluble proteins (**D**), total phenolic (**E**) contents of four cultivars of safflower (*Carthamus tinctorius* L.) foliarly treated with ascorbic acid subjected to water stress conditions (mean ± S.E.).

Proline concentration increased (9.3–9.8%) significantly (*p* ≤ 0.05) in safflower cvs. 16171 and 16246 upon exposure to water deficit conditions. Exogenously applied AsA particularly the 150 mg/L concentration significantly (*p* ≤ 0.001) improved (76.0–93.5%) the proline content in all four cultivars under varying water regimes (Table 1; Figure 3A). All four safflower cultivars were similar in proline accumulation under water deficit conditions and foliar applied AsA.

No significant influence of water stress was observed on the accumulation of glycine betaine (GB) contents in all safflower plants. Of all foliar-applied AsA levels, 150 mg L^−1^ concentration/treatment AsA was found to be significantly effective in increasing the GB contents under water deficit conditions. The response of all safflower cultivars was similar in terms of GB contents to exogenously applied AsA and water deficit conditions (Table 1; Figure 3B).

A significant increase was observed in ascorbic acid contents in all four safflower cultivars due to foliar applied ascorbic acid under both well-watered and water deficit conditions (Table 1; Figure 3C). A significant difference was observed among all cultivars, and among these cultivars, cv. 16207 was higher in AsA content under well-watered regime and cv.16171 under water deficit stress.

Total soluble proteins and total phenolics concentrations increased under water stress conditions. Similarly, AsA exogenously applied at both concentrations significantly enhanced the accumulation of total phenolics and total soluble proteins in all safflower cultivars (Table 1; Figure 3D,E). Of all cultivars, cv. 16246 was superior to other cultivars in total phenolics and cvs. 16171 and 16183 were higher in total soluble proteins accumulation under water deficit conditions.

**Figure 4:**
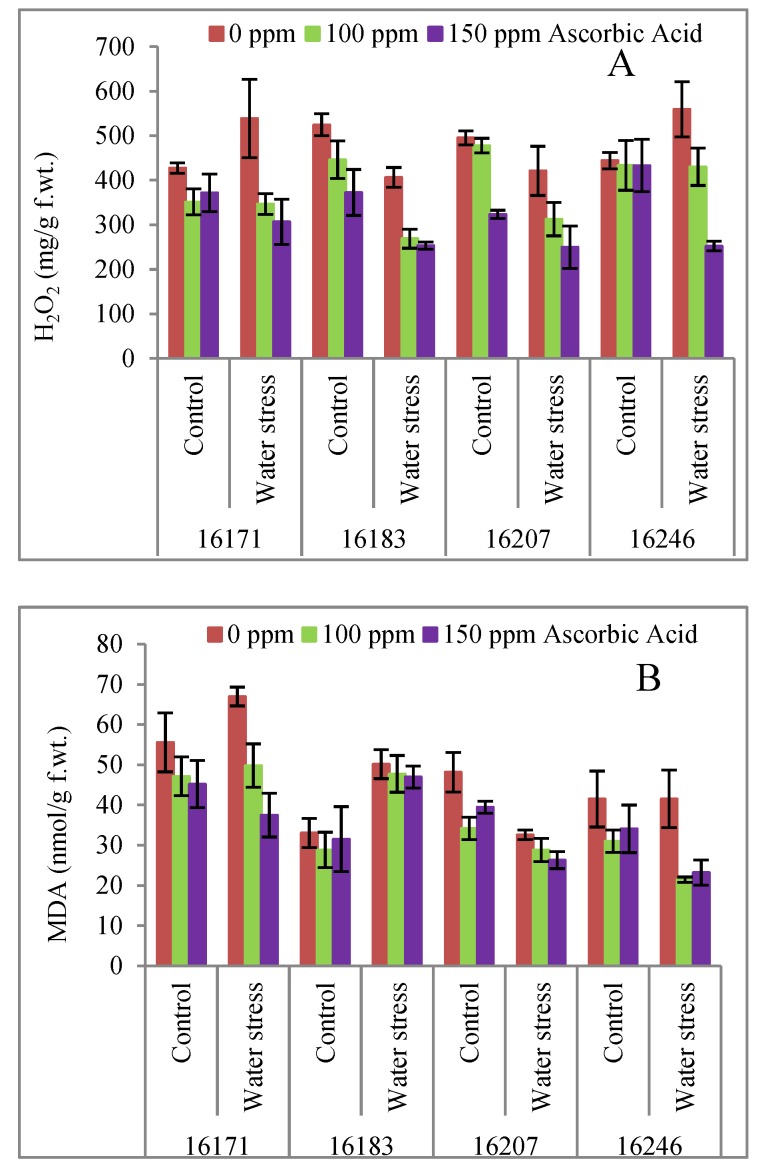
Hydrogen peroxide (**A**) and malondialdehyde (**B**) contents of four cultivars of safflower (*Carthamus tinctorius* L.) foliarly treated with ascorbic acid subjected to water stress conditions (mean ± S.E.).

Under water stress conditions, a significant increase was observed in the concentration of hydrogen peroxide (H_2_O_2_). However, a decrease in H_2_O_2_ accumulation was observed in all safflower cultivars due to exogenously applied ascorbic acid (Table 1; Figure 4A). All safflower cultivars contained similar concentrations of H_2_O_2_ under exogenously applied ascorbic acid and water stress conditions.

An increase was observed in malondialdehyde (MDA) contents in only safflower cultivars 16183 and 16171 due to water stress. However, foliar applied ascorbic acid was effective in lowering the MDA contents. A significant difference was observed among all safflower cultivars due to foliar spray. Of all cultivars, cvs. 16207 and 16246, were lower in MDA contents than the other cultivars, particularly under water deficit conditions (Table 1; Figure 4B).

**Figure 5:**
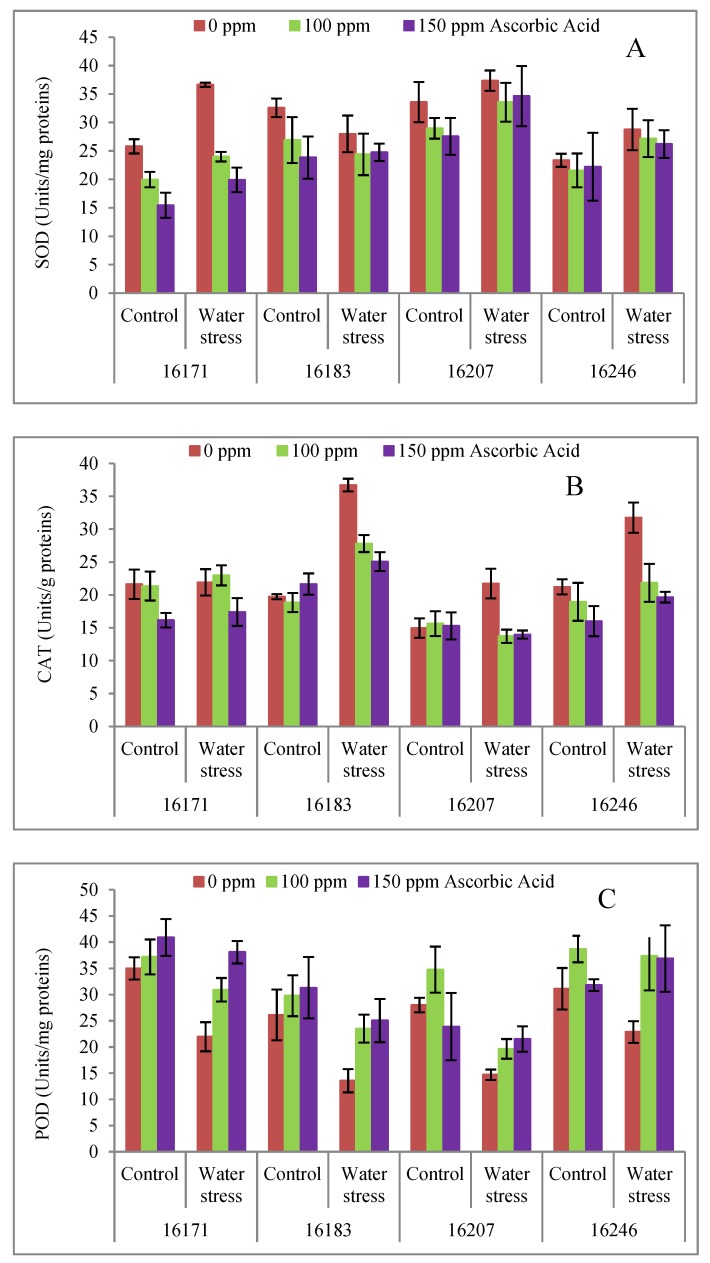
Activities of superoxide dismutase (SOD) (**A**), catalase (CAT) (**B**) and peroxidase (POD) (**C**) enzymes of four cultivars of safflower (*Carthamus tinctorius* L.) foliarly treated with ascorbic acid subjected to water stress conditions (mean ± S.E.).

The activities of CAT, POD and SOD enzymes increased significantly (*p* ≤ 0.001), except that of POD enzyme in cv. 16183 and cv. 16207, wherein it was reduced under water stress conditions. Foliar applied concentrations of AsA (100 and 150 mg L^−1^) were effective in decreasing activities of CAT and SOD enzymes, while increasing activity of POD enzyme in all safflower cultivars under both water regimes. The trend of increase in the activities of the SOD, CAT and POD enzymes was different in all four cultivars. Of all the cultivars, cv. 16183 was better in activity of CAT enzyme, cv. 16246 in POD enzyme and cv. 16207 in SOD enzyme under water deficit conditions (Table 1; Figure 5A–C).

## 4. Discussion

A substantial yield reduction in various economically important crops has been reported due to water deficit stress [30]. In the present study, water stress (60% field capacity) significantly (*p* ≤ 0.001) reduced the plant shoot and root fresh and dry biomass of safflower cultivars (16171, 16183, 16207 and 16246) (Table 1; Figure 1) and a significant inter-cultivar variation was observed in the set of four cultivars. At 100% field capacity, maximum shoot fresh and dry weights were observed in cv. 16171 followed by cv. 16183 and cv. 16246, while under water stress (60% field capacity), the performance of cv. 16207 was better than the other safflower cultivars. However, ascorbic acid application in the present work significantly improved the shoot and root fresh and dry weights especially of cultivars 16171 and 16183 under both control and water deficit regimes (Table 1; Figure 1A–D). Ascorbic acid is a non-enzymatic antioxidant which is believed to be beneficial for protecting plants under water deficit stress [11,16] and it is one of the easily available growth regulators that can be conveniently used by the farmers to induce stress tolerance, including drought tolerance in plants.

It is well known that drought stress can considerably decrease the photosynthetic pigments which result in reduced plant growth and yield [31,32]. Drought stress also leads to the reduction in synthesis of green pigments (chlorophyll contents) resulting in the decreased rate of photosynthesis [32]. In the present study, chlorophyll *a* and *b* contents decreased significantly in all cultivars of safflower. However, maximum reduction in chlorophyll *a* content was observed in cv. 16246 and that of chlorophyll *b* in cv. 16183. Such reduction in chlorophyll pigments may have been due to the enhanced activities of chlorophyllase and peroxidase involved in the breakdown of chlorophyll under stress conditions [33]. In the present study, AsA proved beneficial to reverse the adverse effects of water deficit stress by significantly improving the chlorophyll contents of all four cultivars of safflower. Akram, Shafiq and Ashraf [11] in their extensive review have reported that ascorbate is involved in the breakdown of water in thylakoid lumen, while in stroma it can help to detoxify H_2_O_2_ formed by the action of Cu/Zn-SOD and Fe-SOD.

In response to the water deficit stress, synthesis/accumulation of osmoprotectants in plants is a common phenomenon [34,35,36]. One of the most reported osmoprotectants is leaf free proline which accumulates in plants to mitigate the adverse effects of drought as reported in sweet basil [37], rice [38] and many other crops [35]. In the present study, all safflower cultivars showed enhanced accumulation of proline under water stress. These findings are analogous to what has been observed in several earlier reports [37,38]. Different levels of foliar applied AsA, especially 150 mg L^−1^_,_ significantly improved the proline contents in all four safflower cultivars. The role of ascorbate in proline synthesis is evidenced from some reports. For example, Rana, et al. [39] reported that AsA might be essential for hydroxyproline synthesis, a non-essential amino acid derivative. Likewise, ascorbate is also essential for collagen synthesis, especially the hydroxylation of prolyl residues [39]. Smirnoff and Wheeler [40] in a comprehensive review reported that ascorbate is involved in cell division and expansion of plants as a cofactor for prolyl hydroxylase that hydroxylates proline residues in cell wall hydroxyproline-rich glycoproteins [40]. Despite proline, glycine betaine is also produced in response to water stress and functions as one of the potential osmoprotectants. Glycine betaine can reduce the adverse effects of drought stress by contributing in metabolic pathways, particularly osmoregulation and osmotic adjustments under stress conditions [41]. Foliar applied AsA increased GB concentration in all the four safflower cultivars (Table 1; Figure 3B). The similar behavior of all the four safflower cultivars for proline and GB contents suggested that water deficit stress and exogenously applied ascorbic acid has no connection to the genetic nature of the safflower.

High lipid peroxidation results due to the elevated levels of ROS damaging the plant ultrastructures under water deficit conditions [13,38]. In the present study, high accumulation of H_2_O_2_ and MDA contents were observed in safflower plants subjected to water deficit conditions (Table 1; Figure 4A,B). On the other hand, exogenously applied AsA has played a significant role in lowering the H_2_O_2_ as well as MDA content [41] (Table 1; Figure 4A,B). High oxidative stress is believed to be responsible for H_2_O_2_ generation under abiotic stresses including drought stress [42]. High levels of endogenous AsA maintained antioxidants system to induce tolerance against water deficiency. Moreover, it may modulate the expression of resistant genes and activities of antioxidant enzyme as a bio-signal in signaling pathway thereby minimizing the adverse effects of abiotic stresses [42]. Moreover, in chloroplasts, the role of ascorbate has been suggested during breakdown of water in thylakoid lumen while in stroma to detoxify hydrogen peroxide formed by the action of Cu/Zn-SOD and Fe-SOD [11,43].

In response to water scarcity plants generate both enzymatic and non-enzymatic antioxidants [44]. In the present study, total phenolics were found to be accumulated in high concentrations in all four safflower cultivars under water stress as well as under foliar applied AsA. These results are parallel to what has already been observed in canola [13], *Dracocephalum moldavica* [45], and safflower [17] under water deficit stress. Among safflower cultivars, cv. 16246 was superior to all other cultivars in accumulation of total phenolics in response to water stress as well as foliar-applied AsA. These results suggests that exogenously applied AsA triggers some sort of gene regulatory mechanism to activate such non-enzymatic antioxidants [11,46,47]. In the present study, foliar applied AsA significantly increased the AsA content in all safflower cultivars under water stress and non-stress conditions. Similarly, an increase in AsA content and water stress tolerance have already been observed in different crops, e.g., wheat [48], tomato [49], and canola [13]. Thus, it can be inferred that an increase in AsA level has a significant role in improving plant fresh and dry biomass of all safflower cultivars grown under water-limited conditions. 

Superoxide dismutase (SOD), catalase (CAT) and peroxidase (POD) are important antioxidant enzymes that can protect plants against oxidative stress [9,50]. In the present research, activities of SOD, CAT and POD enzymes increased under water stress except that of POD in cvs. 16183 and 16207, in which it decreased (Table 1; Figure 3A–C). Varying levels of foliar applied AsA resulted in deceased activity of CAT and SOD and enhanced activity of POD in all cultivars under both water regimes. These results agree with earlier reports of enhanced activities of CAT and POD enzymes in wheat fed with AsA [13,50,51]. The varying response of all the cultivars confirmed that the activities of enzymatic antioxidants are possibly due to difference in genetics of these cultivars [52].

## 5. Conclusions

In conclusion, water stress induced an increase in MDA contents, H_2_O_2_, total soluble proteins, total phenolics, leaf free proline and activities of antioxidant enzymes in all safflower cultivars. The exogenously applied AsA appeared to improve plant growth and chlorophyll contents, total soluble proteins, total phenolics, GB, leaf free proline and activity of POD enzyme in all safflower cultivars. We conclude therefore that exogenous application of AsA was effective in improving plant growth and the oxidative defense system under water deficit conditions.

## Figures and Tables

**Table 1:** Mean squares from three-way analysis of variance of data for different morphological and biochemical parameters of safflower (*Carthamus tinctorious* L.) plants treated with foliar spray of ascorbic acid (AsA) under well-watered (control, 100% FC) and water deficit (60% FC) conditions.

Source of Variations	df	Shoot FW	Shoot DW	Root FW	Root DW	Shoot Length
Cultivars (Cvs)	3	37.02 ***	3.208 ***	0.052 ***	0.008 ***	163.4 ns
Drought (D)	1	221.6 ***	15.38 ***	0.034 ***	0.008 ***	6118.4 ***
Ascorbic acid (AsA)	2	26.99 ***	3.477 ***	0.006 ***	0.001 **	1068.5 ***
Cvs × D	3	48.17 ***	4.116 ***	0.0035 **	0.001 **	70.42 ns
Cv × AsA	6	5.13 ***	0.283 *	0.001 ns	0.0001 ns	12.46 ns
D × AsA	2	2.59 *	1.125 ***	0.002 ns	0.0002 ns	11.41 ns
Cvs × D × AsA	6	2.26 ***	0.381 **	0.0007 ns	0.0001 ns	51.76 ns
		**Root Length**	**Chlorophyll *a***	**Chlorophyll *b***		**Proline**
Cultivars (Cvs)	3	19.07 ***	0.316 ***	0.198 ***		0.07 ns
Drought (D)	1	140.3 ***	1.417 ***	0.857 ***		0.68 *
Ascorbic acid (AsA)	2	24.91 ***	0.122 **	0.397 ***		9.83 ***
Cvs × D	3	6.39 ns	0.231 ***	0.036 ns		0.32 ns
Cv × AsA	6	0.813 ns	0.021 ns	0.017 ns		0.14 ns
D × AsA	2	2.08 ns	0.012 ns	0.002 ns		0.83 **
Cvs × D × AsA	6	5.42 ns	0.011 ns	0.031 ns		0.11 ns
		**GB**	**Total Phenolics**	**TSP**	**AsA**	**H_2_O_2_**
Cultivars (Cvs)	3	12.68 ns	67.13 ***	146,428.5 ***	1.72 **	11,391.5 ns
Drought (D)	1	0.10 *	26.96 **	72,409.6 ***	0.695 ns	94,925.5 ***
Ascorbic acid (AsA)	2	145.5 ***	20.62 ***	31,712.8 ***	45.75 ***	199,078.7 ***
Cvs × D	3	80.41 ***	0.402 ns	6380.7 ns	2.558 ***	29,751.7 **
Cv × AsA	6	15.55 ns	4.69 ns	11,800.3 **	0.303 ns	4309.9 ns
D × AsA	2	1.064 ns	2.22 ns	977.5 ns	0.148 ns	31,651.6 *
Cvs × D × AsA	6	4.34 ns	2.702 ns	2211.1 ns	0.336 ns	12,225.1 ns
		**MDA**	**CAT**	**POD**	**SOD**	
Cultivars (Cvs)	3	1545.4 ***	339.2 ***	12.56 ***	379.7 ***	
Drought (D)	1	1.802 ns	470.1 ***	20.65 ***	316.5 **	
Ascorbic acid (AsA)	2	1146.7 ***	253.8 ***	9.95 ***	362.6 ***	
Cvs × D	3	947.5 ***	104.3 **	1.555 ns	88.75 ns	
Cv × AsA	6	137.5 ns	25.48 ns	1.0817 ns	52.19 ns	
D × AsA	2	118.3 ns	109.1 ***	3.812 *	3.153 ns	
Cvs × D × AsA	6	66.22 ns	22.44 ns	0.335 ns	16.25 ns	

ns = non-significant; *, ** and *** = significant at 0.05, 0.01 and 0.001 levels, respectively.
